# A Few Scattering Thoughts on the Contagiousness of Scarlatina

**Published:** 1856-02

**Authors:** Wm. C. Butler

**Affiliations:** East Avon


ART. V.—*A few Scattering Thoughts on the Contagiousness of Scarlatina.*

By WM. C. BUTLER, M. D., East Avon.

It is undoubtedly true, my dear Dr., that the aphorisms of Hippocrates stand in the same relation in one respect, to medical literature and the medical world that the inspired volume does to the ecclesiastical. The different theories that have arisen from the observation of those immutable facts which have withstood the experience of the last two thousand years, can only be equaled by the schisms and isms which surround the pathway of every traveler to that bourne from which none returneth, and must be apparent though he possesses but half an eye.

The ozone theory of the *causa causens of cholera*, of recent date, and the thousand and one prophylactics of tubercle, which deck the pages of medical literature through the long vista of ages, show conclusively, the natural tendencies of certain mental organizations, and the fallacious food which the best of the human family seem so anxious to be fed with. Its parallel can only be found in the heterologous deductions drawn, or professed to be drawn, from the volume of Holy Writ. The crucible of time seems to be wholly forgotten by many theorists, whilst their beautifully drawn fancy sketches seem to them to have been indellibly impressed upon the imperishable page of fame.

Such has ever been the bane of medical literature, and still it may not in justice be so considered. It would seem to be a law of nature, that the antipodes were essential to the due appreciation of either extreme. Let us rather hail, then, with pleasure, these airy flights of the imagination, so long as they have a tendency to draw the attention of medical philosophers to an investigation of these mooted points in medicine.

These wandering thoughts, my dear Dr., were suggested by an article in the September number of the Buffalo Medical Journal, from the pen of my distinguished friend, Dr. Meacham, of Wyoming, (I say friend, for I hold every man to be my friend, until he proves himself an enemy,) whose acquaintance I possess only through the medium of your valuable journal. His writings evince a philosophic mind and a close observance of disease; hence the pleasure of comparing notes with him.

Upon referring to an old *scrawl*, which bears date March, 1840, I find that scarlatina broke out in a family in the south-east corner of my ride, (the diameter of which does not exceed a thousand miles; so that the reader need not let his imagination run beyond the Alleghanies on the south, nor across the lakes upon the north) about the 10th of March of said year. There was nothing peculiar in its visitation, save that there was not another family within a mile but what, from their social relations, would have been more exposed than this. I could not learn, in short, that they ever went away from home, or that any body ever went there. Surely, none, so they averred, had been there in a long while. The disease assumed the simple and anginose varieties.

About the same time, so says the veritable document, March 18, scarlatina broke out in another school district, about five miles directly north of this, which had had no communication with it in any way that could be ascertained, upon a careful inquiry. This we will designate as district No. 2. Ten days subsequently it made its appearance in another district, about the same distance west of district No. 2; and without my being able to learn that either of the three districts had had any human communication in any way. Neither could I learn that it had been endemic there within a few years.

So much for the invasion of the disease, and now for the peculiarities that render it of interest in the case in point. My friend, Mr. B., in whose family the disease first made its appearance, must not pass without my bearing testimony to two exalted qualities, which he possessed in an eminent degree: the first was that he was the best hand to send for a physician I ever knew; a multiplicity of visits never offending him, and if one was disposed to act as nurse as well as doctor, no offense was taken. The second was equal to the first, viz., he was just as good *not* to pay them, as he was to send for them. He also resembled a certain John Rogers, in one respect; having "one small children and *nine at the breast.*" Out of this small number, six of them had scarlatina well marked, and one a scarlet rash upon the mouth and fauces. Four of these were in the first edition; one about half way up the ladder; the other two were the last, and next to the last. Three of six had the anginose variety, and were very sick; the remainder were of the simple form; all passing through their stages with a view of perpetuating the name.

There were five families in this school district, in which the disease did not visit every member; from one to three escaping. In districts numbers two and three, the same facts were noticed, viz., that the disease did not attack every member. In the family of a Mr. Ames, three out of five children had scarlatina at that time; and so on with several others. From these several points it seemed to radiate in several directions, according as it would seem, to some channel of communication, and again were there could not be any discovered.

It spent its fury mostly during the months March and April, and nothing further was seen of it until the month of December, 1841, nineteen months subsequent. It then made its appearance in the family of Mr. Ames, in one of the two children left, after the other epidemic, another being added to the family during the interim. The first child was taken at 6, A. M., and died at 7½, A. M., a well-marked case of the malignant form of the disease—the second was taken at 7, P. M., and died at 11, P. M., also of the same variety. I saw the first case about five minutes before its exit; and the second about an hour, being from home upon the arrival of the messenger. The infant had the simple variety of the disease. It seemed to take, then, as the hunters would say, its back track, visiting those that had escaped before, with what additions had been made to the several communities during the interval. Yea, it reached even the domicil of our friend B., claiming a *set-to* with his *corps* of reserve, or reserve batallion, and again as usual coming off second best. I will not say whether this was a matter of necessity or not, or whether there would have been any more scarlet fever on earth had it attacked the whole phalanx of little B.'s at once, but simply state the facts and let the reader draw his own inference.

Several sporadic cases made their appearance during the next twelve months, when it wholly left the ride until the fall of 1847.

There is one more fact connected with the cases in the family of Mr. B., which is not unworthy of notice taken in connection with the cases of Dr. M. It will not require a very extensive knowledge of mathematics to discover that this illustrious family all told, amounted to near a dozen, and as an excess of furniture was considered an abomination, three beds and a small edition of a trundle bed were considered all-sufficient for them. In these eceptacles old Morpheus made his usual visits to them, as I learned, and two of the milder cases took their usual position, for aught I know, in the loft. One bed being located there, the rest below.

There is still another fact, connected with these cases, which was of peculiar interest to me, and which since that time has been something of a diagnostic in doubtful cases of scarlatina. It was the peculiar livery which all wore that were not implicated in disease. Most of them having some soreness of the throat, amounting to the peculiar sensation in deglutition, and perhaps to an excited imagination, a scarlet rash upon the fauces. Those that escaped well-marked disease in 1840, wore it; and those having it in 1840 wore the "livery" in 1841.

Dr. M. says, taking the contagious view of scarlatina, "The boy, be it remembered, had been subjected to the epidemic influences, if such existed, (this to me is not clearly proved) for a period of many weeks previously, but they had not proved sufficient to institute the malady in his person. It was not till *I slept with him*, after having been daily in the chamber of the lady affected with the malignant type of scarlatina, that the boy's system was contaminated.

"Now let me ask, what are the inferences logically and justly deducible from these premises? Is not one of them this, that the scarlatina of the boy was induced by a specific contagion? Else, why should the boy so long have escaped, and been seized at that particular time? Some may be ready here to put in the plea, "coincidence;" but no reason, no philosophy requires such a plan. The *prima facie* evidence of the existence of the relationship of cause and effect between these two circumstances, is, to say the least, extremely weighty. Is it not proper to infer from this correlation of incidents, that my apparel became laden with a peculiar subtile effluvium from the skin and bronchio pulmonary mucous membrane of my patient, or even that my very body was more or less contaminated with the infinitesimal particles of such a specific poison, and that the boy, from close proximity to the fomites and my own person, was brought within the range of a contagious influence? My own judgment points an unwavering finger to this inference.

"It may be asked, if there was contagion, why had I not been the vehicle of its conveyance to the boy long anteriorly, since I had been in attendance upon scarlatina patients for a considerable time previously? For three reasons, I reply:

"First, because the cases that I had treated before had been of the middle varieties, and the contagion consequently less concentrated and virulent.

"Secondly, because I had not been necessitated to spend as much time over their persons, and thus allow of opportunity for the fixation of the contagious miasm in my dress and person.

"And lastly, because I had not brought the boy into continued, immediate proximity to my own infected body and apparel, as I afterwards did by sleeping with him.

"And here, by way of digression, assuming for the nonce the doctrine of contagion proved, let me say a word in relation to the idea just advanced, that the body of an individual, a physician or a nurse for instance, who has been protected against the peculiar agency of scarlatina by an antecedent attack, may become freighted with the invisible contagion, at least upon its superfices, without suffering its morbific effects, so as to be able to communicate it to those unprotected. The case now under consideration gives evidence to the point—The boy did not suffer from the disease *until after he had slept with me.* I had had scarlet fever in boyhood, and just then furnished not one symptom of an incipient second attack, nor have I since. Indeed, I see nothing unreasonable in the hypothesis. One great characteristic of eruptive fevers, it is well known, is their occurrence, in the main, but once in the life of an individual. The invisible workings of the virus seem to impress upon the system such changes as are ever after prophylactic against the same agent. What this change precisely is, whether, according to Liebig, a process of fermentation, or, according to others, something else, it is of little moment to our present purpose to stop to consider. Suffice it to say that in view of the acknowledged protective virtue of an assault of the disease, I see nothing in the way of the philosophy of the theory indicated above. May not the contagious miasma be fixed upon the cuticle of a protected person, aye, may it not even enter his circulation, without occasioning the disease? I think no one need hesitate long to reply in the affirmative. It is unreasonable to suppose that scarlatina renders the integument and gastro-pulmonary mucous membrane ever after impervious to its peculiar poison, but open and free to every other noxious virus."

Dr. M. will pardon me for not drawing the same deductions from his premises that he has done, which if I understand him right, is something like this:

1st. It requires the virus of the malignant type of the disease to produce the simple form of it when the medium of communication is a protected person, and it requires that the virus must almost be rubbed in at that.

2d. The simple can propagate either of the other varieties when the patient is an unprotected one.

If these propositions were clearly proved it would be quite an acquisition to medical science. But I fancy they are not, save in theory. If the contagious laws which he entertains are correct, why did not every member of the several families above mentioned, have scarlatina at its first visitation? That they were susceptible to the specific effect of the cause, call it what you will, is shown by their having the disease well marked in 1841. Two years ago this present month, I saw a well marked case of scarlatina in a maiden lady of 35, who had followed nursing for many years, and who, up to that time, had always supposed that she had had it in infancy, as she had been exposed to its contagion times without number, without effect previous. Some conclusion is necessarily forced upon us by carefully looking over the number of cases given in the two years. If they prove that scarlatina is contagious, they must of necessity prove its period of incubation, viz., nineteen months. This requires rather a better memory than every disciple of Esculapius possesses, to keep every case distinctly in mind. On the contrary, it would force mediocrê intellects into the erroneous views, perhaps, of non-contagion. You will not be surprised, then, to learn that your humble servant emerged from this field of facts rather a firm believer in the non-contagiousness of scarlatina.

In October, 1847, a man and his wife, and child 18 months old, residing north-west of Geneva, N. Y., visited some friends in Niagara county of this state. During their visit scarlatina was prevailing in the vicinity of their friends, but I could not learn that they saw any cases. On their way home they made the several points hereinafter mentioned, at least; how many more I know not: the first of which I had any cognizance was at the house of a friend in the town of Caledonia. 2d. Avon do. 3d. West Bloomfield. 4th. In the town of Canandaigua.

From these several points which I took the pains to examine personally, scarlatina broke out in the several families at which they stopped, and radiated from them to a greater or less extent in several directions, depending upon the amount of food left for it by other visitations of disease, more than anything else, according to the information which I could obtain. Thus far there was nothing peculiar about the disease, only that the several cases in each type, simple and anginose, were very mild. Yes, one thing more — its period of incubation did not extend to nineteen months — also that their child was passing safely through the simple form of the disease. Some two months subsequent to this, I learned still more of the progress of the case. On his arriving home scarlatina broke out among his relations about a week after, assuming the malignant form of the disease. It spread rapidly and fearfully. A mother, who was my informant, told me that four weeks previous she was rich in the possession of five beautiful children, three of them she had consigned to one grave, and two slept side by side in another, and if ever a mother's countenance told of deep. intense and indiscribable suffering, at which language would seem but an idle mockery, it was hers. Heaven grant that I may never witness another such as hers. She said it had almost depopulated one school district; that it had seemed to show the most virulence in those localities which were most subject to the low types of fever. But as this, though not without interest, is not relative to the object of the present paper, we will let it pass.

Here, then, was a well-marked case of scarlatina which had its origin from scarlatina, and which generated scarlatina wherever it could be traced, almost or quite as surely as variola does variola, or any other form of contagious disease does its like.

It is said that the late lamented T. Romeyn Beck once related the following case to his class, in one of his absent minded paroxysms:

He said a like case (one in medical jurisprudence) was once submitted to the learned Zaccheus for his decision; who, after examining the testimony on each side, carefully came to the conclusion that there was as much evidence on one side as there was upon the other, *if not a little more.* Such was, then, my situation, minus the learned.

Are there, then, no logical deductions to be drawn from the above series of facts. I fancy there are, and some of them would seem to be these:

1st. Of the laws which govern many forms of epidemic influence in the present state of medical science we are most profoundly ignorant.

2d. From the social relations which exists between Jew and Gentile, in this country, a thousand and one channels of communication may exist which the closest scrutiny may not for the time being develop.

3d. There may be, and probably are, peculiar conditions of the animal economy influenced by the various causes which operate upon it under all circumstances, that render it susceptible to the influence of the virus of scarlatina at one time and not another, and also give type to it.

4th. There may be, and are, as is shown in my own case, peculiarities in the *tout ensemble*, which render the possessor an object unworthy the notice of this dire scourge for a series of years, without exonerating him from an attack at some subsequent time.

That the above positions are correct, is shown by comparing its type and the various grades of that type in different localities, and in the same locality under different influences; or, in other words, the description of scarlatina in one locality may or may not be a correct one in another. That this is correct, the following description given me by an intelligent physician, who resided in one locality for a long series of years, will demonstrate. Should any one for a moment imagine that he was not conversant with the medical literature of the age, he will have the pleasure of imagining what no one who had the pleasure of his acquaintance would for one moment believe.

Several years since while on a visit to my parents, who reside in Onondaga county, I met the late Dr. Samuel Healy, of Syracuse, who had then practiced medicine there nearly or quite half a century, and of whom it is enough to say that he held the same relation as a practitioner of medicine to Syracuse and vicinity, that Dr. Ellwood has to Rochester. He was a close observer of disease, and weighed well all its modifying influences. He said, speaking of scarlatina, that sixty years ago now, 1855, it made its first appearance in that vicinity, and was known under the common name of black tongue — that death took place often before ulceration of the throat commenced — and that no distinct and characteristic rash made its appearance until some time subsequent to this. He thinks there was but one type of the disease, and that the malignant, for several years. Death and black tongue were, during this period, nearly synonymous terms. Fifty years since it made its appearance again, under the cognomen of canker rash, having the peculiar eruption of scarlatina, and losing, to a great extent, its synonym. Near forty years since it made another visitation, in a much milder form, and from this cause a great war of words arose among the learned M. D.'s as to its identity. Previous to this time the rash had been confined to the face, neck and upper portion of the chest, although the cuticle had desquamted over the whole system. At this time the rash extended more generally, and there was less distinction of the soft parts of the mouth and fauces, and the matter was compromised, by giving the name of canker rash to such cases as had ulceration of the mouth and throat, and scarlatina to those cases in which this did not obtain to a great extent. It will readily be discovered, that a complicated case of *canker rash and scarlatina often occurred requiring more skill in the management*, with designing men, than either singly! Between forty-five and thirty years since, sporadic cases made their appearance in all the varieties of the disease for the first time. Dr. H. was a firm believer in the non-contagiousness of scarlatina, having witnessed facts without number similar to those above mentioned, and never having had the disease himself. Few men, perhaps, have done the amount of consultation business in western New York that he has, and I again repeat, that few equaled him as a profound thinker.

Some thirty years ago was the first your humble servant ever knew of scarlatina, when it broke out in the family of an uncle, who was a near neighbor, and who lost two children with the disease.

My earliest impressions were received from a Dr. Cowles, of Marcellus, who made the astounding discovery that the two fatal cases were canker rash instead of scarlatina. From that day to this I have seen more or less of it, but as yet have never had it. Why, I know not; but do know that it is not from want of exposure in nursing, &c. I have known many that have been exposed many times in nursing, &c., and still have the disease well marked at a subsequent period.

That we have scarlatina as one of our most formidable diseases many times, and many times one of the simplest, is no longer a matter of question. That the believers for or against its contagiousness may be numbered by thousands, is of no importance to mankind in general, or the profession in particular. But that there are rational causes in every community that must facilitate its ravages or modify its fatality, are questions, the solution of which is of vital importance to the whole human family. Ambition should ask not another laurel for his brow who gives us this solution.

There are few diseases known to the general practitioner whose pathological complications are more numerous and important than in this. And none, I regret to say, that baffles the skill and judgment of the most scientific, equal to it; and none, in my humble opinion, in which we can form so incorrect an opinion of medical capabilities of our neighboring practitioners as the success or want of success, they meet with in treating this disease. Its sequelæ, equally numerous and complicated, are frequently of little moment, and again far more fatal than the disease itself; but thanks to the progressive nature of our noble profession, it is being better understood and fast being disrobed of its fatality.

That we have much yet to learn with regard to the ætiology and the modifying influences of this truly protean malady, few will question who are so unfortunate as to have much of it to treat. That we have much more still to learn to combat successfully the poisonous effects of the virus upon the fluids of the system, few, I think, will endeavor to gainsay. For my own part, I have watched with no little anxiety to see what disposition the crucible of experience was disposed to make with the mur. tinct. ferri in erysipelas, in hopes that it would meet the anxieties of the profession, and that it, or some other similar compound, would be found equally as efficacious in the poison of scarlatina.

That this is the true path for future research in this disease, I have long and firmly believed; nor do the developments of pathological anatomy in its sequæle lessen the hope that this desideratum will yet be fully realized by the profession.

